# Modelling the Effect of Viruses on Insect Survival: Using a Second-Order Phase Transition Model to Describe Time–Effect and Dose–Effect Relationships Using Entomopathogenic Viruses as an Example

**DOI:** 10.3390/insects16101023

**Published:** 2025-10-03

**Authors:** Vladislav Soukhovolsky, Anton Kovalev, Olga Tarasova, Dmitry Kurenshchikov, Yuriy Tokarev, Daria Kharlamova, Yuriy Akhanaev, Sergey Pavlushin, Vyacheslav Martemyanov

**Affiliations:** 1 V.N. Sukachev Institute of Forest, Siberian Branch of Russian Academy of Sciences, Krasnoyarsk 660036, Russia; 2 Krasnoyarsk Scientific Center, Siberian Branch of Russian Academy of Sciences, Krasnoyarsk 660036, Russia; 3 Department of Ecology and Nature Management, Siberian Federal University, Krasnoyarsk 660041, Russia; 4 Institute of Systematics and Ecology of Animals, Siberian Branch of Russian Academy of Sciences, Novosibirsk 630091, Russia; 5 Institute of Water and Ecological Problems, Far East Branch of Russian Academy of Sciences, Khabarovsk 680000, Russia; 6 All-Russian Institute of Plant Protection, sh. Podbelskogo 3, Pushkin, St. Petersburg 196608, Russia; ytokarev@vizr.spb.ru; 7 Scientific Center of Genetics and Life Sciences, Sirius University of Science and Technology, Sirius 353340, Russia

**Keywords:** insects, viruses, dose, mortality, temporal dynamics, lethal time, model, second-order phase transitions

## Abstract

The present study examines the effect of viruses on forest insects depending on the virus dose. Two model approaches are used to quantify the effect of viruses on insect survival. Both approaches describe the processes of virus exposure to insects within the framework of the second-order phase transition model, which is well known in theoretical physics. The first approach examines the temporal dynamics of larval survival at a given dose of virus exposure. This dependence is characterized by the time–effect curve. In this case, the lethal time of exposure LT100 is the time required for the death of all larvae in the experiment at a given dose *D* of exposure. The second approach describes the relationship between the proportion *q_r_* of larvae that survived a fixed time *T_c_* after the start of the experiment and the dose *D* of virus exposure. This dependence is characterized by the dose–effect curve. The experiments tested the effect of two different viruses—nucleopolyhedrovirus (NPV) and cypovirus (CPV)—on such insect species as *Lymantria dispar* L., *Manduca sexta* L. and *Loxostege sticticalis* L. It was shown that the proposed models of second-order phase transitions very accurately (with coefficients of determination of the models close to *R*^2^ = 0.95) describe experiments on studying the effect of different virus strains on insect survival. The proposed models turned out to be useful for assessing the effectiveness of different virus strains against insect pests. Since the parameters of the second-order “dose–time” and “dose–effect” phase transition models are related to each other, it is possible to reduce the number of measurements of virus–insect interaction due to the relationship between these parameters, and instead of *n* observations of insect dynamics over time depending on the dose of exposure, the basic parameters characterizing the “virus–insect” interactions can be accurately estimated using only one measurement. It appears that the proposed model can be used to calculate the effect of toxic agents on the population of victims for a wide variety of toxicant species and populations. A sharp reduction in the labor intensity of experiments to assess the toxicity of certain toxicants on animal populations will simplify and reduce the cost of testing the response of living objects to toxicants.

## Simple Summary

This study explores how viruses can control forest insects by examining how the amount of virus and exposure time affect insect death rates. The goal was to create simple mathematical models to predict how well viruses work against insect pests. Researchers tested two entomopathogenic viruses (nucleopolyhedrovirus and cypovirus) and found that their models accurately predict insect deaths (with a high accuracy of 95%) depending on treatment doses. These models use ideas from physics to describe how viruses impact insects, similar to how physical systems change states. By using just one experiment, the models can estimate how effective different virus strains are at killing pests. The findings are valuable because they offer a way to estimate effective doses using a low amount of tested doses compared to the commonly used probit- or logit-based analysis approaches.

## 1. Introduction

Today, the effects of viruses are considered as a tool of biological control capable of reducing insect pest populations [1,2,3].

One of the first parameters estimated in the research of strains of entomopathogenic viruses, as well as any other entomopathogen, proposed as potential control agents is the dose/concentration of the entomopathogen needed to initiate an economically significant protective effect. Another critical parameter is the time required for virus to cause insect mortality. The virus replicates in the host organism for 5 to 14 days depending on its genetic features before reaching the terminal stage of pathogenesis and causing the insect to die [4,5]. This is particularly important for viruses of the Baculoviridae family, which have a complex replication cycle in the host, leading to longer ontogenesis of the host with an increase in the duration of feeding [5,6]. Now, the effectiveness of entomopathogenic viruses is mainly evaluated experimentally, for example, in bioassays, where the dose–effect relationship is determined by testing serial dilutions of the virus [7,8]. At least five concentrations of the viruses and 30 larvae per dose are used in experiments. The effectiveness of viruses is mostly assessed using probit or logit models or, for example, the Weibull model [8]. However, these models are nonlinear, and many experiments with different serial dilutions need to be carried out to find model parameters. Research involving numerous assays to evaluate the effectiveness of the virus brings up the question of whether the number of experiments aimed at determining the parameters of the dose–time or dose–effect curves can be decreased and, ultimately, reduced to a single experiment with only one exposure dose.

In experiments estimating the effect of viruses on insect larvae, the percentage *p* of the killed larvae is usually determined after a standard time period *T_c_* [9]. However, the use of the dose–effect relationship gives rise to the following key question: how much time, *T_c_*, should pass between the onset of the exposure to the virus and the evaluation of the effect? As the time between the onset of the exposure and the effect directly depends on the dose [10], the duration of the experiment is usually reduced by using the dose leading to the death of 50% of the insects [11], that is, *p* = 0.5. At *T_c_* → 0 and *p* → 1, we deal with the extremely effective strain of the virus. Usually, however, *T_c_* > 0, and the death rate *p* of the insects is increased with an increase in the standard time period *T_c_*. At the same time, it is not always convenient to choose the *T_c_* as the period over which all larvae are sure to die, because such experiments may last too long. An arbitrary choice of *T_c_* may result in the following situation: at a nonlinear mortality rate *v*(*t*) of the larvae, the selected *T_c_* will correspond to the state in which, at a low *T_c_*, the percentage of surviving larvae *q*(*t*) will be rather high, i.e., *p* << 1, whereas if *T_c_* is increased, the mortality may rise, and ultimately, *p*(*T_c_*) → 1. Then, if the selected *T_c_* is too low, the effectiveness of the viruses will be underestimated.

Quite often, within the framework of the dose–effect model, researchers, instead of data analysis, consider temporal survival curves *q* = 1 − *p*(*T*) [12]. In such cases, the effect of the virus can be characterized by the time span, *T_r_*, between the exposure onset at time *t*_0_ and the death of all larvae in the experiment at time *t_r_.* Then, *Tr* = LT100 = *t_r_* − *t*_0_ can be characterized as the lethal time (LT), the time of the death of all larvae. The lower LT100 is at a certain exposure dose, the more effective the virus is. Even this approach, though, does not solve the problem of selecting the time span for observing the effect of the virus on the insects. It may take too long for all larvae to die, and moreover, not all larvae may be killed by a specific virus strain. In that case, it will not be possible to wait until all larvae die in the experiment.

Analysis of survival is, in the general case, based on construction of statistical models, in which effect *y* (response of the ecosystem) is the function of independent variables (*x*; *t*), where *x* is the impact level and/or other exogenous or endogenous factors affecting the lifetime of targets *t* [12,13,14,15,16]. A variety of methods have been developed by statisticians in many areas, such as medicine, engineering, and biological sciences, for the proper analysis of lifetime or time–response data [17,18,19]. Both parametric and nonparametric procedures have been described, and issues such as normality, censoring or truncation of data, and correlation of data points have been addressed.

For a particular impact process, the task is to choose the most acceptable model which capable of determining not only the average values of the system’s response to impacts but also statistical characteristics of the impact (standard deviation, standard error). The choice of the model is usually based on how well any given model approximates the data obtained in the experiment. Different models may turn out to be optimal for exposure to different virus strains, and then, various types of models with different free parameters should be compared. However, it would be good to obtain not approximations, but a generalized theoretical model that would not only be able to describe all possible experiments but also provide an explanation for the observed processes and predict the effect of viruses on insects, reducing the number of experiments to the minimum. Such a model could standardize procedures for evaluating the effects of viruses on insects and substantially decrease the time and effort required to assess the impact of viruses on insect pests.

Larval mortality dynamics are obviously determined by the initial concentration (dose *D*) of the virus, its replication rate (determined by the virus replication ratio and resistance of the larvae), and the critical virus density at which the larva dies. If, in the experiment, the initial individual dose for each larva is the same, the virus has the same replication rate and critical density, and all larvae are equally resistant to the impact of the virus, meaning all larvae must die simultaneously. No such results are observed in actual experiments, which suggests that the properties of the virus strain and larvae vary, resulting in various larval death rates. Unfortunately, no express forecast of virus growth parameters and larval resistance characteristics can be made, and thus, their interaction is assessed using the indirect parameters of average virus dose and population death rates [16].

The present study proposes describing mortality of the insects exposed to viruses using a survival curve model taking into account qualitative differences in the responses of the insects during their exposure to viruses, as opposed to models with a uniform effect of the virus on insects. The insects’ response can be considered as the second-order phase transition, and this model will have a number of advantages over the classical probit and logit models. The possibility of using a small number of serial dilutions of the virus for constructing the model will be assessed, and the critical (minimal) time span necessary for determining mortality correctly will be estimated to provide a basis for constructing a working model. Both parameters are critical for biologists, and if the doses or observation periods are reduced, considerably less time and effort will be needed to conduct assays, or if the insects are not readily available, an opportunity to test them will be created.

## 2. Materials and Methods

### 2.1. Modeling the Effect of Viruses on Insects

Viruses infect and kill insects. However, the effectiveness of viruses in killing larvae is largely determined by the dose of the virus. Two model approaches can be used to quantify the effect of viruses on insect mortality. One approach is to consider temporal dynamics of larval survival at a preset virus exposure dose. This approach is characterized by the time–effect curve. The lethal time LT100 is the duration of the exposure to the virus required for all larvae in the experiment to die, i.e., *q*(LT100) = 0. The other approach describes the relationship between the percentage of the larvae *q_r_* that survive some definite time *T_c_* after the start of the exposure and the virus dose *D*. This relationship is characterized by the dose–effect curve.

We propose a basic model for describing the effect of viruses on insects as an analog of the model of second-order phase transitions in physical systems [20]. This model is used because larvae are not immediately killed by the virus, and the infected group of the insects can exist in several phases: the phase in which all larvae are alive, the phase in which larvae are dying, and, finally, the phase in which all insects are killed (sometimes, the death of all insects is not observed in the experiment). In terms of theoretical physics, when a physical system is exposed to an external factor, the description of the process should involve the introduction of a Hamiltonian, which describes the energy of the physical system as dependent on the type of interactions in it [20]. However, for a complex physical system (more complex than the hydrogen atom), it is extremely difficult to write such a Hamiltonian (or potential function *G* of the system) because of the complexity of interactions of the system components [21]. For these conditions, it was suggested that processes in the physical systems that are exposed to external factors and experience phase transitions (qualitative changes) should be described using equation *G*(*q*, *X*), which describes the value of potential function *G* of the system as dependent on the value of the order parameter *q*, characterizing some macroscopic properties of the study system, and external factor *X* [20,22]. As the true form of the constraint equation for these values is not known, this relation is described using expansion into Taylor series of function *G*(*q*, *X*) in powers of *q*:

(1) $$G=G_{0}+a(X-X_{r})q^{2}+bq^{4}$$

where *G*_0_, *a*, and *b* are some constants, and *X_r_* is the critical value of the external factor at which phase transitions occur in the system.

If *G* → min, Equation (1) has two solutions. According to the first solution, when the external factor is greater than some critical value *X_r_*, the value of the order parameter at which $\frac{dG}{dq}=0$ is equal to zero. If the value of the external factor is *X* < *X_r_*, the order parameter is determined by the *X* value, and this constraint equation is written as follows:

(2) $$q^{2}=\left\{\begin{array}{l} 0,\;X\geq X_{r}\;\\ \frac{a(X_{r}-X)}{2b}=\alpha_{1}-\beta_{1}X,\;X<X_{r} \end{array}\right.$$

where *α*_1_ = *aX_r_*/2*b*; *β*_1_ = *a*/2*b*.

**Figure 1:**
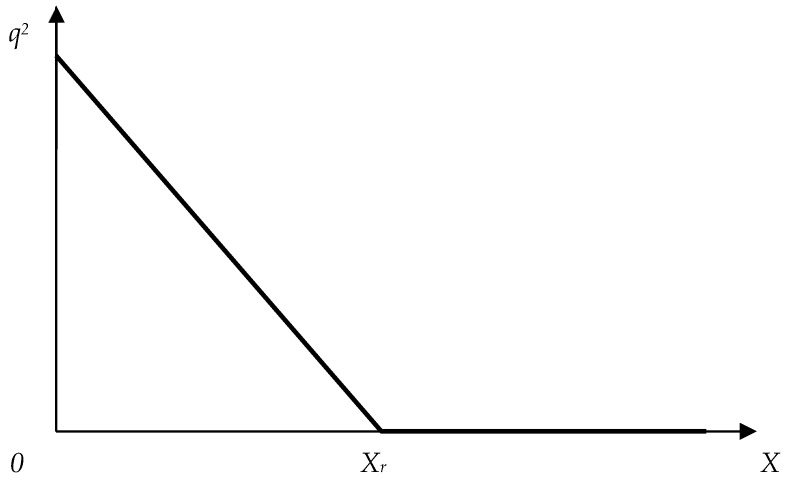
Theoretical relationship between the order parameter *q* and the exposure characteristic.

Figure 1 shows the relationship between the order parameter *q* and the exposure characteristic according to Model (2).

Up to a certain value of factor *X_r_*, the squared order parameter linearly declines as *X* is increased (Figure 1). When *X_r_* reaches its critical value, the order parameter becomes equal to zero.

Can Equations (1) and (2) be used to describe the behavior of a biological object exposed to external dose *D*? To be able to describe this process within the frameworks of Models (1) and (2), two differences of biological systems from physical ones should be taken into account. First, in a physical system, the response to exposure to an external factor is immediate. In biological systems, though, there is a delay in the response of the system to external impact, and hence, there is a so-called lag phase, when, for some time *T*_0_ after the onset of the exposure, the order parameter *q* characterizing the state of the system does not change. Second, under certain conditions, the order parameter does not reach the value of *q* = 0 by the end of the experiment, i.e., not all larvae die.

Taking into account these properties of biological systems, in the equation of the temporal dynamics of larval mortality caused by viruses, virus dose *D* should be introduced as its initial concentration, and the order parameter *q* should be determined as the percentage of larvae that have survived over time *T* of exposure to dose *D*. Then, using (1) and (2) and taking into account specific biological properties, temporal dynamics of insect mortality caused by exposure to virus dose *D* can be described by the following equation:

(3) $$q^{2}(T,D)=\left\{\begin{array}{l} 1,\;T\leq T_{0}(D)\\ \alpha_{1}-\beta_{1}T,\;T_{0}(D)<T\leq T_{r}(D)\\ q_{r}^{2},\;T>T_{r}(D) \end{array}\right.$$

where *T*_0_ is the duration of the lag phase of the effect when no larval death is observed. *T_r_* is the critical time point regarding larval exposure to the virus, at which point larvae stop dying, and *q_r_* is the percentage of live larvae by the end of the experiment. *α*_1_ and *β*_1_ are coefficients; $\beta =\frac{\partial q^{2}}{\partial T}$ is the susceptibility of the insects to the effect of the virus.

The critical value of order parameter *q_r_* characterizes the percentage of live larvae at the end of the experiment. If *q_r_* = 1, the tested virus strain does not affect the insects. If all larvae exposed to dose *D* die over time *T_r_* of the experiment, *q_r_*(*D*) = 0, and *T_r_* = LT100. The results of the experiment with different exposure doses can be used to construct *D*, dependence of LT100, i.e., the dose–time curve.

Thus, with this approach, the effect of viruses on insects is described by Equation (3) with the following free parameters: duration of the lag phase *T*_0_, susceptibility of insects to virus exposure *β*_1_, and critical time LT100 of exposure of larvae to the virus.

An equation analogous to Equation (3) relates the effect *q* to the exposure dose *D*. For the relation *q* = *g*(*D*, *T_r_*), linked to the percentage *q* of the insects surviving by the end of the experiment and the exposure dose *D*, the following equation can be written:

(4) $$q^{2}(D)=\left\{\begin{array}{l} 1,\;D\leq D_{0}\\ \alpha_{2}-\beta_{2}\ln(D+1),\;D_{0}<D<D_{r}\\ q_{r},\;D>D_{r} \end{array}\right.$$

where *D*_0_ is the virus dose at which insects do not die, and *D_r_* is the dose at which all larvae die.

If, at dose *D_r_*, all larvae die, then *q_r_* = 0, and doses greater than *D_r_*, at which *q* = 0, can be termed as overkill doses; α_2_ and *β*_2_ are constants. If *α*_2_ > 1, at dose *D* ≤ *D*_0_, *q*^2^ = 1. $\beta_{2}=\frac{\partial (q^{2})}{\partial (\ln(1+D))}$ is the susceptibility of insects to exposure to dose *D*.

From (4), it follows that at *D* ≤ *D*_0_, *q* = 1, i.e., all insects remain alive. In exactly the same way, *q* = 1 if *β*_2_ = 0, that is, the virus does not affect the insects.

These two representations of relationships between the exposure dose and its effect are clearly interrelated, but they characterize different aspects of how viruses affect insects.

In what follows, the models of the effects of viruses on insects described above will be used to discuss experimental results on mortality of various species of insect larvae exposed to different virus strains.

Apparently, specialists—entomologists—will find it somewhat difficult to become used to this proposed approach. However, firstly, it is very difficult to accurately translate physical ideas into entomological language. Therefore, perhaps this should not be explored. The history of science is well known for cases idea “flowing”. A classic example is C. Darwin’s use of economic and demographic ideas from T.R. Malthus in constructing the theory of natural selection in biology. Another example is the “flowing” of the economic idea of resource distribution by V. Pareto into linguistics (G.K. Zipf). In all these and many other similar situations, it turned out that the ideas put forward to solve problems in one area of science could be effectively used in a completely different subject area, and this should be taken into account.

### 2.2. Material

#### 2.2.1. The Infection of *Lymantria dispar* by LdMNPV Isolates

As a source of empirical data, we used one of the frequently used herbivore–entomopathogenic virus systems, spongy moth *Lymantria dispar* L.–*Lymantria dispar* multiple nucleopolyhedrovirus (LdMNPV). Infection of *L. dispar* larvae with LdMNPV isolates was carried out by the droplet feeding method [23]. Newly molted 2nd-instar larvae were offered a droplet (0.5 µL) of a suspension consisting of 100 mg/mL sucrose, 0.01 mg/mL red dye, and occlusion bodies (OBs). The LdMNPV doses were 5, 15 50, 150, 500, and 5000 OBs/larva. These baculovirus dose ranges led to larval mortalities between 5 and 95%. The larvae that ingested the whole droplet were placed in a 350 mL ventilated plastic container with the host plant. The larvae that failed to consume the whole droplet were excluded from the bioassay. Four to three replicates (containers) were used with 10 larvae per container for each LdMNPV dose (40 or 30 larvae/dose). Several strains of LdMNPV were used for this study. These strains were used for infection of several *L. dispar* populations from Europe (Krasnodar) and Asia (Novosibirsk and Khabarovsk) (Table 1).

#### 2.2.2. The Infection of *Lymantria dispar* by DsCPV-1

The droplet feeding method was also used to infect *L. dispar* larvae by *Dendrolimus sibiricus* cytoplasmic polyhedrosis virus (DsCPV-1). Each individual newly molted 2nd-instar *L. dispar* larva was offered a droplet (0.5 µL) of 100 mg mL^−1^ sucrose with 0.01 mg mL^−1^ red dye and one of five doses of DsCPV-1. In particular, 0.5, 5, 50, 500, and 5000 OBs per larva were used. The larvae were starved for approximately 4 h prior to inoculation. Larvae in a control group were fed with virus-free water droplets. Larvae that ingested the whole droplet were placed in a 350 mL ventilated plastic container with a host plant. The insects were reared under laboratory conditions at a constant temperature (23 °C) and with a natural daylight regime. The larvae that failed to consume the whole droplet were excluded from the bioassay. Five or four replicates were used with 10 larvae per container for each DsCPV-1 dose.

#### 2.2.3. The Infection of *Manduca sexta* by DsCPV-1

Eighty 2nd-instar larvae per dose of the virus were infected. Infection was performed in groups: 20 larvae/0.5 L plastic container and 4 containers/dose. As the larvae aged, each group was divided between two containers (i.e., after the 4th instar, in each container, there were 10 larvae). The larvae were infected with cypovirus isolated from dead larvae of *Dendrolimus sibiricus*, which was recently described by our team [24]. The following concentrations of DsCPV-1 were used: 10^4^, 3 × 10^4^, 10^5^, 3 × 10^5^, 10^6^, and 10^7^ polyhedra/mL. A suspension of CPV polyhedra was topically applied onto the surface of the nutrient medium (0.5 mL/20 larvae). In the control group, 0.5 mL of distilled water was used. Concentrations, rather than doses, were used because of the specific biological development of *M. sexta* on an artificial diet and the impossibility to apply a drop feeding approach. Dead larvae were counted every day. The dead larvae were examined using qPCR as described elsewhere [25].

#### 2.2.4. The Infection of *Loxostege sticticalis* by DsCPV-1

Beet webworm *L. sticticalis* 2nd-instar larvae were infected using DsCPV-1 concentrations of 6 × 10^5^, 2 × 10^6^, 6 × 10^6^, 1.7 × 10^7^, 5.3 × 10^7^, and 1.6 × 10^8^ polyhedra/mL. The virus suspension of a known concentration was transferred to a Petri dish. Burdock leaves were immersed in a Petri dish with a known concentration of the virus for 30 s. Five leaves per concentration were used. There were 10 larvae per one treated leaf. Larvae were allowed to stay at the treated leaves for 24 h. The control-group larvae were offered untreated leaves. The leaves used in the bioassay were size-selected.

## 3. Results

**Figure 2:**
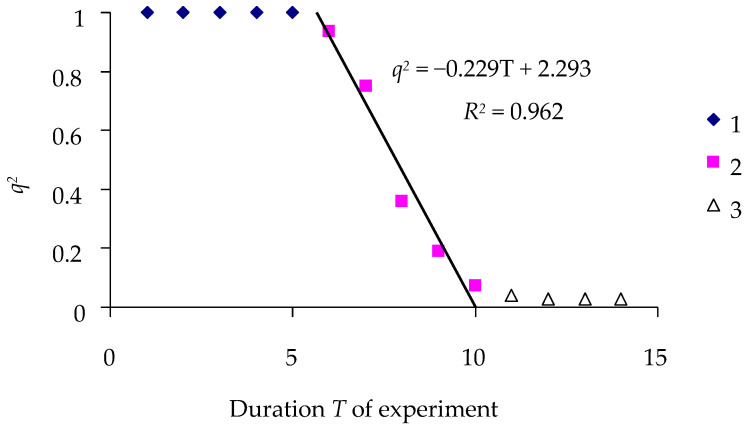
Temporal dynamics of the percentage *q* of surviving insects exposed to the virus LdMNPV-27/0 at a dose of *D* = 50 OBs/larvae (Krasnodar population). 1—lag phase; 2—dying period; 3—post-mortal period.

To illustrate the calculation of the time–effect model, Figure 2 demonstrates the effect of the LdMNPV-27/0 virus (Baculoviridae) at a dose of *D* = 50 OBs/larva of the spongy moth from the Krasnodar population, according to Model (3).

The experimental data are in good agreement with Model (3) (Figure 2), and after over 10 days of exposure, almost all larvae in the experiment died. The coefficient of determination *R*^2^ = 0.959 of equation *q*^2^ = *α*_1_ − *β*_1_*T* is very close to 1 at *T* < *T*_0_, at which *q* = 1, and at *T* > *T_r_*, at which *q* = 0. The data in Figure 2 suggest that *T*_0_ = 5 days, *β*_1_ = 0.232, and LT100 = 2.3056/0.232 = 9.93 days.

Table 2 lists results of calculations for experiments estimating the effects of LdMNPV strains on spongy moth populations in Krasnodar (Caucasus), Japan, and Siberia.

In all experiments, mortality dynamics were described by Model (3) very well, and the coefficients of determination for Equation (3) were close to 1 (Table 2).

The proposed models are nonlinear; however, the methods of linear regression analysis are applicable in this case, since these models can be brought to a linear form by transforming the parameters. For example, the exponential model is considered linear since, by taking its logarithm to the natural base, we can obtain a linear form of the model [26,27]. In this case, to evaluate the regressions under consideration, one can use the OLS methods and estimates such as the coefficient of determination.

Equation (3) can be derived for larvae exposed to viruses at dose *D*, and the critical lifetime LT100(*D*) of the larvae at this exposure dose can be estimated. Then, for all doses of the virus used in the experiment, the following constraint equation can be obtained for the series {LT100(*D*)} and {ln (*D* + 1)}:

LT100 = *A* − *B* ln (*D* + 1) (5)

where *A* and *B* are constants.

**Figure 3:**
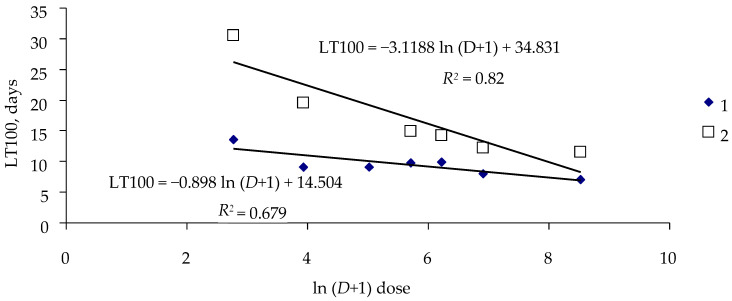
Dose–critical time relationship (Khabarovsk spongy moth population): 1—virus strain LdMNPV-BibJ; 2—virus strain LdMNPV-KR.

Figure 3 shows the dose–critical time relationship for the Khabarovsk spongy moth population exposed to virus strains LdMNPV-BibJ and LdMNPV-KR.

Parameters *A* and *B* of Equation (5) characterize the effect of a certain virus strain on the population. Table 3 presents statistical parameters of Equation (5) for two strains to which the Khabarovsk spongy moth population was exposed.

All parameters of Equation (5) for different virus strains are statistically significant, and coefficients *A* and *B* for different strains differ from each other at a level of *p* = 0.05 (Table 3).

**Figure 4:**
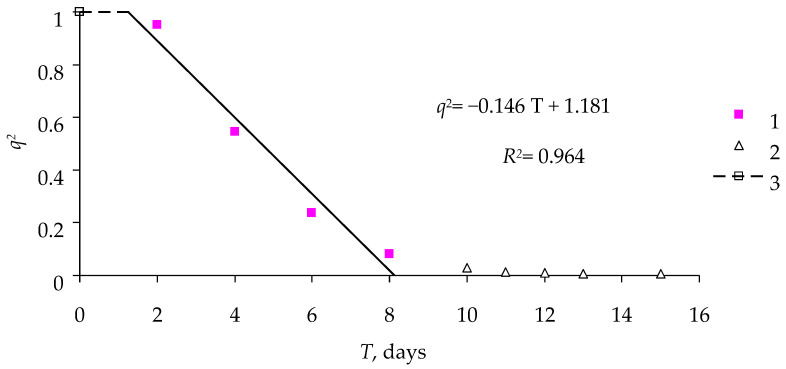
Temporal dynamics of mortality of *M. sexta* larvae exposed to DsCPV-1 at a concentration of 10^8^ polyhedra/mL: 1—the phase after the onset of exposure; 2—the phase of the death of all larvae; 3—lag phase.

Similar temporal dynamics of the effect of the virus were observed for another entomopathogenic virus, DsCPV-1, representing another family (Spinareoviridae), in the experiment with the *M. sexta* larvae (Figure 4).

Table 4 lists the parameters of Model (3) for the effect of DsCPV-1 on *M. sexta*.

Model (3) describes the mortality dynamics of *M. sexta* exposed to cypoviruses (Table 4) very well. The coefficient of determination *R*^2^ for this equation is close to 1 and significant at a level no lower than *p* = 0.018; the coefficients of Equation (3) are significant as well.

How closely are the parameters of the model of second-order phase transitions interconnected? Table 5 shows relationships between parameters of exposure dose *D*, duration of lag phase *T*_0_, and timespan LT100 for experiments with spongy moth populations from different habitats exposed to various virus strains.

A rather strong relation (with coefficients of determination *R*^2^ in many cases close to 1) is observed between values of *q_r_* and ln (*D* + 1) for different virus strains and various spongy moth populations (Table 5). Thus, the final percentage of surviving larvae *q_r_* can be estimated from the exposure dose *D*.

Similar results were obtained in calculations of the proposed model for experiments in which 2nd-instar *L. dispar* larvae were infected by the DsCPV-1 virus using the drop feeding method (data cited from [24]). The following virus concentrations were used: *A* = 10^7^ polyhedra/mL, *B* = 10^6^ polyhedra/mL, *C* = 10^5^ polyhedra/mL, *E* = 10^4^ polyhedra/mL, *F* = 10^3^ polyhedra/mL, and *G* = control. The effect was estimated by the percentage of the larvae that had survived by Day 15 of the experiment. The results of the experiments are presented in Table 6.

**Figure 5:**
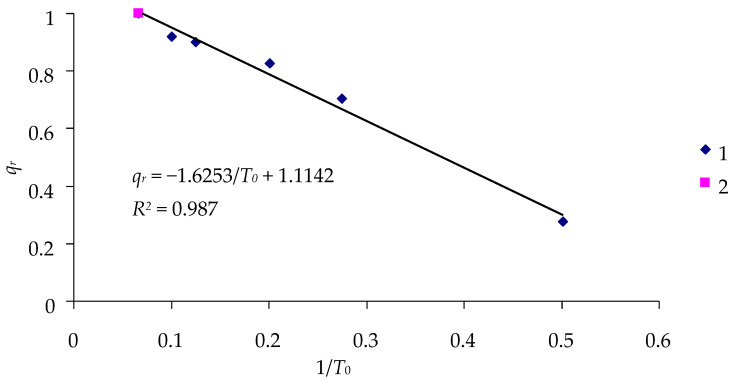
Relationship between the inverse duration of the lag phase 1/*T*_0_ and the percentage of surviving insects *q_r_* in the 15-day experiment with *L. dispar* larvae infected by the DsCPV-1 virus: 1—treatments; 2—control.

The data in Table 6 can be used to estimate the relationship between the duration of the lag phase *T*_0_ and the percentage of live insects *q_r_* at the end of the experiment (Figure 5).

Again, the 1/*T*_0_ value can provide a practically exact assessment of the effectiveness of DsCPV-1, based on the percentage of surviving insects at the end of the experiment. With the experiment lasting 15 days and no larvae dying in the control, 1/*T*_0_ = 0.067, and *q_r_* = 1 (point 2 in Figure 5). Then, the slope of the curve and the absolute term in the constraint equation for *q_r_* and 1/*T*_0_ in Figure 5 can be determined with high accuracy based on the results of the experiment with a single exposure dose, and the effects of other doses of the cypovirus can be assessed by approximating the equation in Figure 5.

If the calculation is performed using the 1/*T*_0_ data in the control with the given duration of observation *T* and survival of all control larvae (i.e., *q_r_* =1) and the data of one experiment with the exposure dose of *D* = 10^6^ polyhedra/mL, then for *T* = 15 days, the constraint equation *Q_r_* = −1.4248/*T*_0_ + 1.095 can be written for these two points, where *Q_r_* is the calculated value of the effectiveness of exposure. Then, other values of the effectiveness of exposure to the virus, *Q_r_*, can be determined and compared with the observed values of *q_r_* (Table 7).

The error, Δ*q_r_* = (*q_r_* − *Q_r_*), of cypovirus effectiveness calculation based on the results of the experiment with the *B* 10^6^ polyhedra/mL calculation only for one dose, *A* 10^7^, is somewhat different from the experimental *q_r_* values (Table 7). At other doses, differences in the effects in the experiment and in calculations do not exceed 0.03, which has no practical influence on evaluating the effectiveness of the cypovirus.

**Figure 6:**
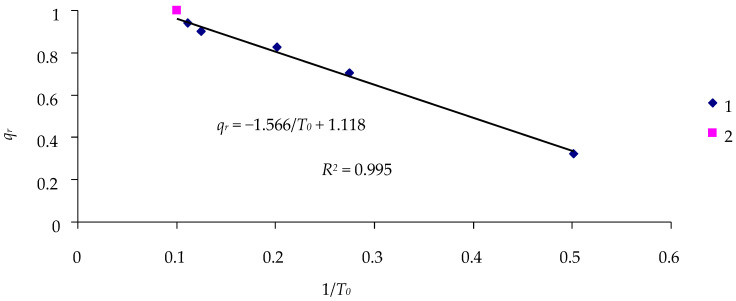
Relationship between the inverse duration of the lag phase 1/*T*_0_ and the percentage of surviving insects *q_r_* in the 10-day experiment with *L. dispar* larvae infected by the DsCPV-1 virus: 1—treatments; 2—control.

Similar results of calculating the effectiveness of the cypovirus can be obtained if the experiment lasts 10 days (Figure 6).

If the experimental duration is *T* = 10 days, the constraint equation *Q_r_* = −1.566/*T*_0_ + 1.118 can be written for points with *D* = 0 and *D* = 10^6^. Then, the effectiveness of the virus, *Q_r_*, over the 10-day observation period can be determined (Table 8).

The calculated values of virus effectiveness are closer to the experimental ones, and the absolute difference Δ*q_r_* between the experimental and calculated values is no greater than 0.02 (Table 8).

**Figure 7:**
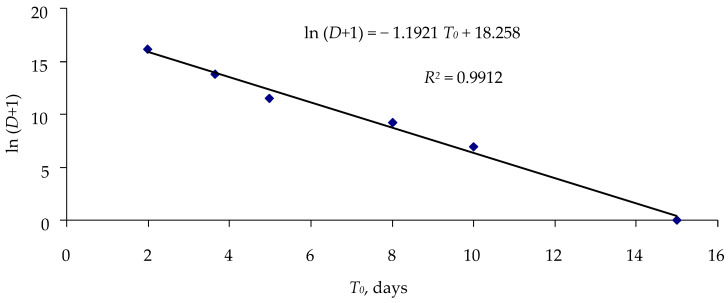
Relationship between lag phase *T*_0_ and log concentration ln (*D* + 1) of virus DsCPV-1 infecting *L. dispar* larvae.

Thus, evaluation of the effect of cypoviruses on survival of larvae can be based on the results of a single (!) 10-day experiment with a single exposure dose. The percentage of surviving larvae can be estimated using the simplified constraint equation for 1/*T*_0_ and *q_r_*, and through setting some values of 1/*T*_0_, one can find the corresponding ln (*D* + 1) value for this value of 1/*T*_0_ from the equation in Figure 7.

**Figure 8:**
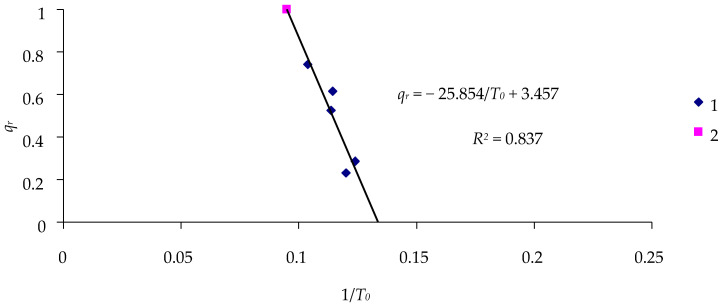
Relationship between the inverse duration of the lag phase 1/*T*_0_ and the percentage of surviving insects *q_r_* in a 16-day experiment with Siberian *L. dispar* larvae infected by the LdMNPV: 1—treatments; 2—control.

**Figure 9:**
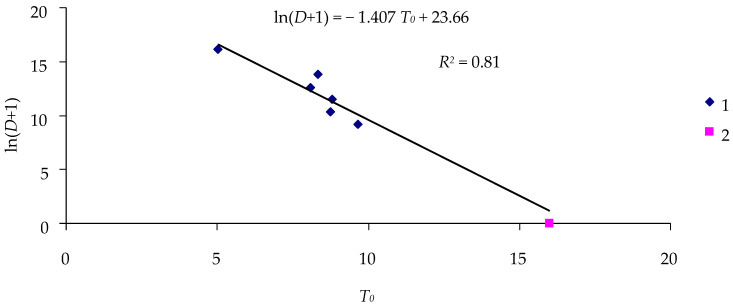
Relationship between the duration of the lag phase *T*_0_ and log dose ln (*D* + 1) of the LdMNPV infecting Siberian *L. dispar* larvae: 1—treatments; 2—control.

Similar calculations can be performed using the data in Table 2 for exposure of Siberian *L. dispar* larvae to LdMNPV (Figure 8 and Figure 9).

Table 9 presents calculations of the effectiveness of the LdMNPV in the Siberian spongy moth population, similar to the above calculations of the effectiveness of the DsCPV-1 infection of insects.

These calculations, which are also based on the experiment with one concentration of the virus, are sufficiently close to the calculations for all experiments.

However, not all experimental procedures used to study the effects of viruses on insects can be simplified. Specific effects are observed in experiments with the beet webworm *Loxostege sticticalis*. The results of these experiments differ substantially. Even without exposure to the virus, *L. sticticalis* larvae die at ln (*D* + 1) = 0; thus, at *D* = 0, *q* will be less than 1. Even without infection, when *D* = 0 and ln (1 + *D*) = 0, *q*^2^ = 0.53, although the theoretical value is *q*(*D* = 0) = 1.

This effect might be caused by spontaneous (stochastic) death of the larvae, unrelated to the exposure to the DsCPV-1. The data on insect mortality in the control show this quite clearly (Table 10).

**Figure 10:**
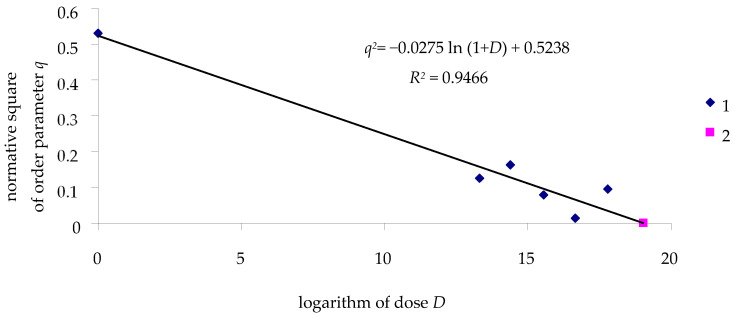
The dose–effect relationship for *L. sticticalis* larvae infected by DsCPV-1: 1—experiment; 2—calculated value, ln (1 + LD100), characterizing the log dose at which all larvae die.

It is usually assumed that in experiments conducted to study the effect of viruses on insects, the only reason why insects die is exposure to baculoviruses. If this were the case, at *D* = 0, all (or at least almost all) larvae in the control should survive. However, in experiments with the beet webworm, on average, about 20% of the larvae died, even in the control (Table 10). Only in one experiment did no larvae die in the control. Assuming that stochastic mortality of *L. sticticalis* larvae was not dependent on the exposure dose of the DsCPV-1, the mortality of the larvae in the treatments can be interpreted as a result of the additive effects of the virus and stochastic factors. To calculate the impact of viruses, in this case, it is possible to select the average of the two minimal values of *q*^2^ at a given dose for estimating *q*^2^ at this dose. Here, we consider the maximal contribution of stochastic effects to larval mortality. The dose–effect relationship for *L. sticticalis* exposed to the DsCPV-1 is shown in Figure 10.

Thus, ln (1 + LD100) for the treatment group is 19.04. To summarize, experiments aimed at studying the effects of viruses on insects should take into account not only the absolute mortality of the experimental insects but also the causes of their deaths in order to obtain an assessment of the “pure” effect of viruses on insects.

## 4. Discussion

Is it correct to use the model of second-order phase transitions to describe the effects of viruses on insects, and why are commonly accepted models such as logit and probit analysis and the Weibull equation worse for this purpose [12,13,14,15,16]? An important factor is that for continuous models of the logit and probit types, the probability of the death of the larvae exposed to viruses is not equal to zero in experiments of any duration (even short durations). In actual experiments, though, at any viral dose, there is a lag phase, and larvae do not die for some time after the onset of exposure, i.e., classical models are not valid for this time span.

Researchers designing experiments aimed at evaluation of the effects of viruses or any other pathogens on insects face the challenge of minimizing expenditures on bioassays of larvae. In the limiting case, survival of larvae infected by the virus is studied in experiments with *k* doses of the virus, in which *s* observations of the state of the larvae last time *T*. Then, experimental results can be used to determine the values of *T*_0_, *β*_1_, *q_r_*, and LT100 and the dose–effect relationship. In order to minimize the expenditures, the number of different doses of the virus, *k*, the number of *s*, and the duration of observation *T* should be reduced.

In any case, it is necessary to estimate the duration of the lag phase *T*_0_, as after the lag phase, the relationship between the *q*^2^ and *T* values is linear and characterized by the equation *q*^2^ = *α*_1_ − *β*_1_*T*. In the simplest case, coefficients *α* and *β* can be determined from two values of *q*^2^(*T*): $\overset{⌢}{\beta }1=\frac{q_{2}^{2}-q_{1}^{2}}{T_{2}-T_{1}}$ and $\overset{⌢}{\alpha }1=q_{1}^{2}-\overset{⌢}{\beta }1T_{1}$. The calculated values of $\overset{⌢}{\beta }1$ and $\overset{⌢}{\alpha }1$ can be used to estimate LT100. It is certainly impossible to determine the standard errors of the calculated parameters by using this approach, but in complete experiments, these errors will be small and will not affect the qualitative evaluation of the effectiveness of the virus.

Broadly speaking, analysis of interactions between viruses and insect larvae is not the ultimate goal of research, but rather a means to assess the ways to control pest populations using a biological insecticide. The assessment of the effectiveness of controlling pest populations can be considered in association with the parameters of the model of second-order phase transitions. In the system of pest population control, two tasks can be addressed: (1) quickly suppressing a certain percentage of the pest population in order to reduce the current level of damage caused by insects to plants and (2) reducing the minimum reproduction rate of the pest population (even if the current population cannot be suppressed quickly). During the lag phase, larvae are not yet damaged, and, evidently, they are still feeding on plants. This is particularly characteristic of the exposure to Baculoviridae viruses, which cause generalized infection. Therefore, viruses with low *T*_0_ values should be used to increase the mortality level of insects. The higher the value of susceptibility *β*_1_ of larvae to the viral infection, the quicker these larvae will die and the less food they will consume. Thus, the goal of quick suppression of pests can be achieved either by selecting virus strains/species with low *T*_0_ values and high *β*_1_ values or by using adjuvants that reduce the *T*_0_ value [24,28].

To decrease future pest population density, one must ensure that the *q_r_* value in Model (3) tends to zero even if the conditions of quick suppression are not fulfilled. Then, if the number of insects is small, their productivity and reproduction rate will be low, and the pest population density will be lower in the next season.

## 5. Conclusions

The present study showed that the use of models of phase transitions for describing the effect of viruses on insects provides a rather accurate evaluation of the dose–effect curve, based on the results of an experiment with a single dose/concentration of the virus with no other factors of larval mortality. What makes it possible to reduce the number and scale of the experiments aimed at studying the susceptibility of insects to viral infections? It seems that the cause of the success is that, in contrast to the standard methods of probit and logit analysis, models of phase transitions include a priori equations with prescribed variables that describe the interaction process, and these equations are linear. It appears that the proposed model can be employed for preliminary estimation (if the smallest possible number of doses is used) of the effects of different toxic agents on various prey populations. Owing to the dramatic decrease in the labor and time required to perform experiments aimed at estimating the toxicity of different substances for biological objects, assays testing response of living organisms to toxicants will become simpler and less costly.

## Figures and Tables

**Table 1:** The scheme of infection of *L. dispar* populations (rows) by LdMNPV strains (columns).

*L. dispar* population	LdMNPV infection used in this study, tested on *L. dispar* population			
Novosibirsk (Siberia)	LdMNPV-27/0	LdMNPV-KR	LdMNPV-KG	LdMNPV-BibJ
Krasnodar (Caucasus)	LdMNPV-27/0	LdMNPV-KR	LdMNPV-KG	LdMNPV-BibJ
Khabarovsk (Far East)		LdMNPV-KR		LdMNPV-BibJ

**Table 2:** Parameters of the time–effect model for strains of *LdMNPV* used to infect *L. dispar*.

Virus Strain and Concentrations (Doses)	ln (1 + *D*)	*β*_1_	*α*_1_	*R* ^2^	*T_r_*	*q_r_*	ln *q_r_*
Krasnodar							
10^4^ (5)	9.210	0.089	1.7379	0.98	8.291	0.617	−0.482
3 × 10^4^ (15)	10.309	0.138	2.142	0.900	8.275	0.550	−0.600
10^5^ (50)	11.513	0.104	1.918	0.97	8.827	0.694	−0.365
3 × 10^5^ (150)	12.612	0.126	2.054	0.98	8.365	0.550	−0.600
10^6^ (500)	13.816	0.222	2.745	0.93	7.860	0.36	−1.022
10^7^ (5000)	16.118	0.075	1.267	0.93	3.560	0.154337	−1.869
Japan							
10^4^ (5)	9.210	0.066	1.451	0.999	6.833333	0.664	−0.410
3 × 10^4^ (15)	10.309	0.303	3.66	1	8.778878	0.576	−0.553
10^5^ (50)	11.513	0.371	4.339	1	9	0.476	−0.743
3 × 10^5^ (150)	12.612	0.124	1.846	0.98	6.822581	0.401	−0.914
10^6^ (500)	13.816	0.185	2.377	0.96	7.443243	0.344	−1.068
10^7^ (5000)	16.118	0.115	1.476	0.95	4.13913	0.166	−1.796
Siberia							
10^4^ (5)	9.210	0.189	2.823	1	9.646	0.743	−0.297
3 × 10^4^ (15)	10.309	0.1563	2.366	0.90	8.740	0.617	−0.482
10^5^ (50)	11.513	0.238	3.096	0.91	8.807	0.524	−0.646
3 × 10^5^ (150)	12.612	0.199	2.606	0.93	8.070	0.287	−1.248
10^6^ (500)	13.816	0.285	3.374	0.999	8.330	0.233	−1.456
10^7^ (5000)	16.118	0.151	1.759	0.91	5.026	0.084	−2.474

**Table 3:** Statistical parameters of Equation (5) for two strains to which the Khabarovsk spongy moth population was exposed.

Strain	Parameters	Values	Standard Error	*t*-Test	*p*
LdMNPV-BibJ	*A*	14.50	1.61	8.98	0.00029
	*B*	0.90	0.28	3.26	0.0226
	*adjR* ^2^	0.62			
	F-test	10.6			
LdMNPV-KR	*A*	5.70	0.97	5.89	0.0042
	*B*	−0.64	0.16	−3.94	0.017
	*adjR* ^2^	0.74			
	F-test	15.5			

**Table 4:** Parameters of Model (3), time–effect, for describing interactions between *M. sexta* and DsCPV-1.

Parameters	Values	Std.Err.	*t*-Test	*p*-Value
concentrations 0				
*α*_1_	1.044	0.024	43.044	0.000000
*β*_1_	0.014	0.003	−5.410	0.000639
*T_r_*, days	74.601			
*T*_0_, days	4.3			
*adjR* ^2^	0.760			0.00069
F-test	29.3			
Dose 10^7^				
*α*_1_	1.045	0.034	30.828	0.000000
*β*_1_	0.038	0.004	−10.497	0.000006
*T_r_*, days	27.535			
*T*_0_, days	1.2			
*adjR* ^2^	0.924			0.000006
F-test	110.2			
Dose 3 × 10^7^				
*α*_1_	1.021	0.029	35.448	0.000000
*β*_1_	0.081	0.004	−21.969	0.000001
*T_r_*, days	12.564			
*T*_0_, days	0.3			
*adjR* ^2^	0.986			0.000001
F-test	482.6			
Dose 10^8^				
*α*_1_	1.181	0.109	10.794	0.008475
*β*_1_	0.146	0.020	−7.282	0.018339
*T_r_*, days	8.118			
*T*_0_, days	1.3			
*adjR* ^2^	0.950			0.018
F-test	53.0			

**Table 5:** Relationships between parameters of the model of second-order phase transitions for different virus strains to which spongy moth populations from different locations were exposed.

Parameters	Novosibirsk	Novosibirsk	Novosibirsk	Khabarovsk	Khabarovsk	Krasnodar	Krasnodar	Krasnodar	Krasnodar
	LdMNPV-KR	LdMNPV-BibJ	LdMNPV-27/0	LdMNPV-BibJ	LdMNPV-KR	LdMNPV-BibJ	LdMNPV-27/0	LdMNPV-KR	LdMNPV-KG
*T*_0_ = *A*1 − *B*1 ln (*D* + 1)									
*A*1	12.76	12.44	13.44	10.32	14.04	10.95	10.26	9.94	11.08
*B*1	−1.10	−1.01	−1.08	−0.83	−1.58	−0.83	−0.75	−0.65	−0.82
*R* ^2^	0.68	0.62	0.80	0.97	0.89	0.74	0.63	0.45	0.60
*q_r_* = *A*2 − *B*2 ln (*D* + 1)									
*A*2	0.97	0.95	0.98	0.88	1.31	0.71	0.72	0.92	0.92
*B*2	−0.06	−0.06	−0.08	−0.09	−0.18	−0.06	−0.07	−0.10	−0.07
*R* ^2^	0.88	0.98	0.96	0.71	0.98	0.64	0.72	0.92	0.79
ln (*D_r_* + 1)	15.16	14.89	11.75	9.61	7.31	11.82	9.86	9.34	13.57

**Table 6:** Results of experiments with 2nd-instar *L. dispar* larvae infected by DsCPV-1.

Experiment	ln *D*	*b*	*a*	*R* ^2^	*q_r_* ^2^	Days	*q_r_*	*T_r_* = *a*/*b*
*A*	16.12	0.173	1.345	0.987	0.076	2.00	0.275	7.78
*B*	13.82	0.094	1.342	0.904	0.495	3.64	0.70	14.28
*C*	11.52	0.093	1.46	0.999	0.681	4.97	0.825	15.78
*E*	9.21	0.098	1.195	1	0.810	8.00	0.90	12.26
*F*	6.91	0.058	1.052	0.96	0.846	10.00	0.92	18.14
*G*	0					15.00	1	

**Table 7:** The effectiveness of the virus calculated using the data of one experiment lasting *T* = 15 d with a dose *D* = 10^6^ polyhedra/mL.

Concentrations, pol/mL	ln (*D* + 1)	*T*_0_	1/*T*_0_	*q_r_*	*Q_r_*	Δ*q_r_*
*G* control	0	15	0.066667	1	-	-
*A* 10^7^	16.12	2.00	0.50	0.28	0.38	−0.10
*B* 10^6^	13.82	3.64	0.27	0.70	-	-
*C* 10^5^	11.51	4.97	0.20	0.83	0.81	0.02
*E* 10^4^	9.21	8.00	0.13	0.90	0.92	−0.02
*F* 10^3^	6.91	10.00	0.10	0.92	0.95	−0.03

**Table 8:** Cypovirus effectiveness values calculated from the data of one experiment, *T* = 10 days, with the dose *D* = 10^6^ polyhedra/mL.

Experiment	ln (*D* + 1)	*T*_0_	1/*T*_0_	*q_r_*	*Q_r_*	Δ*q_r_*
*G*	0	10	0.1	1	-	
*B*	13.81551	3.638298	0.274854	0.703377	-	
*A*	16.1181	1.995954	0.501014	0.322727	0.333413	−0.011
*C*	11.51294	4.972973	0.201087	0.825	0.803098	0.022
*E*	9.21044	8	0.125	0.9	0.92225	−0.022
*F*	6.908755	9	0.111111	0.94	0.944	−0.004

**Table 9:** Effectiveness of the exposure to the cypovirus calculated using the data of one experiment with the virus concentration of *D* = 3 × 10^5^ polyhedra/mL (150 polyhedra/larva).

*D*	ln (*D* + 1)	1/*T*_0_	*q_r_*	*Q_r_*	Δ*q_r_*
0	0	0.095	1	-	
3 × 10^6^	12.612	0.124	0.287	-	
10^4^	9.210	0.104	0.743	0.786	−0.043
3 × 10^5^	10.309	0.114	0.617	0.521	0.096
10^6^	11.513	0.114	0.524	0.543	−0.019
10^7^	13.816	0.120	0.233	0.382	−0.149

**Table 10:** Mortality of the *L. sticticalis* larvae in the control (*D* = 0) by the end of the experiment.

Experiment	% Mortality
1	0.125
2	0.125
3	0.250
4	0
5	0.333
Average	0.174

## Data Availability

All data presented in this article are included within Results section of this article.
